# From Phantoms to Patients: Improved Fusion and Voxel-Wise Analysis of Diffusion-Weighted Imaging and FDG-Positron Emission Tomography in Positron Emission Tomography/Magnetic Resonance Imaging for Combined Metabolic–Diffusivity Index (cDMI)

**DOI:** 10.3390/diagnostics14161787

**Published:** 2024-08-16

**Authors:** Katharina Deininger, Patrick Korf, Leonard Lauber, Robert Grimm, Ralph Strecker, Jochen Steinacker, Catharina S. Lisson, Bernd M. Mühling, Gerlinde Schmidtke-Schrezenmeier, Volker Rasche, Tobias Speidel, Gerhard Glatting, Meinrad Beer, Ambros J. Beer, Wolfgang Thaiss

**Affiliations:** 1 Department of Nuclear Medicine, University Hospital Ulm, 89081 Ulm, Germany; 2 Siemens Healthineers AG, 91052 Erlangen, Germany; 3 Experimental Cardiovascular Imaging (ExCaVI), Department of Internal Medicine II, Ulm University Medical Center, 89081 Ulm, Germany; 4 Department of Diagnostic and Interventional Radiology, Ulm University Medical Center, 89081 Ulm, Germany; 5 Section Thoracic and Vascular Surgery, Department of Cardiac and Thoracic Surgery, Ulm University Medical Center, 89081 Ulm, Germany; 6 Department of Internal Medicine II, Ulm University Medical Center, 89081 Ulm, Germany; 7 Center for Translational Imaging (MoMAN), Ulm University, 89081 Ulm, Germany; 8 Surgical Oncology Ulm, i2SOUL Consortium, Albert-Einstein-Allee 23, 89081 Ulm, Germany; 9 Core Facility PET/MR, Medical Faculty, Ulm University, 89081 Ulm, Germany

**Keywords:** PET/MRI, registration, DWI, image fusion, magnetic resonance imaging, FDG

## Abstract

Hybrid positron emission tomography/magnetic resonance imaging (PET/MR) opens new possibilities in multimodal multiparametric (m2p) image analyses. But even the simultaneous acquisition of positron emission tomography (PET) and magnetic resonance imaging (MRI) does not guarantee perfect voxel-by-voxel co-registration due to organs and distortions, especially in diffusion-weighted imaging (DWI), which would be, however, crucial to derive biologically meaningful information. Thus, our aim was to optimize fusion and voxel-wise analyses of DWI and standardized uptake values (SUVs) using a novel software for m2p analyses. Using research software, we evaluated the precision of image co-registration and voxel-wise analyses including the rigid and elastic 3D registration of DWI and [18F]-Fluorodeoxyglucose (FDG)-PET from an integrated PET/MR system. We analyzed DWI distortions with a volume-preserving constraint in three different 3D-printed phantom models. A total of 12 PET/MR-DWI clinical datasets (bronchial carcinoma patients) were referenced to the T1 weighted-DIXON sequence. Back mapping of scatterplots and voxel-wise registration was performed and compared to the non-optimized datasets. Fusion was rated using a 5-point Likert scale. Using the 3D-elastic co-registration algorithm, geometric shapes were restored in phantom measurements; the measured ADC values did not change significantly (F = 1.12, *p* = 0.34). Reader assessment showed a significant improvement in fusion precision for DWI and morphological landmarks in the 3D-registered datasets (4.3 ± 0.2 vs. 4.6 ± 0.2, *p* = 0.009). Most pronounced differences were noted for the chest wall (*p* = 0.006), tumor (*p* = 0.007), and skin contour (*p* = 0.014). Co-registration increased the number of plausible ADC and SUV combinations by 25%. The volume-preserving elastic 3D registration of DWI significantly improved the precision of fusion with anatomical sequences in phantom and clinical datasets. The research software allowed for a voxel-wise analysis and visualization of [18F]FDG-PET/MR data as a “combined diffusivity–metabolic index” (cDMI). The clinical value of the optimized PET/MR biomarker can thus be tested in future PET/MR studies.

## 1. Introduction

The introduction of magnetic resonance imaging (MRI) and positron emission tomography (PET) has greatly improved medical imaging over the past few decades.

MRI uses magnetic fields and radio waves to non-invasively produce detailed anatomical images, with a particular strength for soft tissues [1]. As a non-invasive method that does not involve radiation, MRI is widely used for diagnostic purposes and for monitoring treatment response. In particular, the combination of anatomical information with functional parameters, such as diffusion-weighted imaging (DWI) or dynamic contrast-enhanced imaging (DCE), allows for its use in oncological imaging, for example, in characterizing liver lesions [2,3,4]. Briefly, DWI measures the diffusion of water molecules in tissue. It is particularly sensitive to the random motion of water molecules, which can vary between normal and abnormal tissue. In oncology, it can help differentiate between benign and malignant tumors based on differences in cell density [2,5,6].

PET, on the other hand, involves the injection of a radioactive tracer into the body, which is absorbed by organs and tissues, and [18F]-Fluorodeoxyglucose (FDG)-PET has been used in the past decades to evaluate glucose metabolism in various organ systems and tumors [7,8]. 

While both MRI and PET alone provide critical insight into various health conditions, the combination of both modalities to benefit from excellent tissue contrast, DWI, and metabolic imaging has long been desirable. After solving complex technical issues, a combined hybrid imaging modality with magnetic resonance imaging (MRI) and positron emission tomography (PET) in PET/MRI was ready for clinical use more than ten years ago [9,10,11,12]. One advantage of using a hybrid imaging modality combining magnetic resonance imaging (MRI) and positron emission tomography (PET) in PET/MRI is the simultaneous acquisition of different imaging parameters, which allows for the parallel acquisition of anatomic, metabolic, and functional information. In comparison to sequential acquisitions conducted using disparate scanners, this approach is demonstrably superior, particularly in regions exhibiting high intrinsic movement, such as the thorax and upper abdomen [13,14]. However, the disparate acquisition methodologies employed for dedicated MRI sequences pertaining to diffusion, perfusion, and high-resolution anatomy, in addition to PET acquisition, have resulted in inherent variabilities in image resolution and temporal resolution. Furthermore, minor regional discrepancies between MRI sequences and PET acquisitions, as well as slight movements in the bowel due to bowel movements or cardiac activity, resulted in inconsistencies in the precise overlay of images. Furthermore, distortion artifacts can be pronounced in some sequences, for instance, due to bowel gas in DWI. These constraints impede the optimal overlay of the acquired images. In recent times, several techniques have been proposed with the aim of overcoming these limitations. The use of breath-hold and triggered examinations has been demonstrated to reduce the variability of breathing position. Post-processing approaches have concentrated on rigid and non-rigid transformations of images with the objective of overcoming the resolution discrepancies between MRI sequences and PET images [15,16,17].

The combination of diffusion imaging and [18F]FDG-PET has been extensively studied over the past few years. This is because the combination of cellularity and metabolic activity has been shown to reflect viability and response to treatment in various cancers after chemo- and radiation therapy in several studies [14,18,19,20,21,22,23,24,25]. While several studies employed a sequential combination of PET and computed tomography (PET/CT) and MRI, recent research has demonstrated the advantages of a combined PET/MRI system. Nevertheless, these limitations are compounded by the lack of an exact voxel-wise co-registration, as the standard overlay provided by the manufacturers is limited, especially for diffusion images and PET images. In the context of this study, an exact voxel-wise co-registration would be desirable in order to characterize thoracic lesions by both uptake in [18F]FDG and DWI characteristics.

The objective of this study was to implement an optimized approach for the co-registration of MR diffusion images and PET images in phantom measurements and in bronchial carcinoma patients who underwent simultaneous PET/MR imaging for staging, with the aim of improving voxel-wise analyses. Secondly, we conducted a precision analysis of the image fusion and visualization co-registration using research software at the voxel level.

## 2. Materials and Methods

### 2.1. Phantom Generation

As a surrogate for the tissue, we employed a 1% (*w*/*v*) agar gel with various fractions of sucrose to regulate the diffusivity of the material [26]. Three-dimensional (3D)-printed phantoms with cavities were constructed and filled with agar–sucrose gels of varying compositions to simulate different types of tissue. To simulate a lesion with high FDG uptake, [18F]FDG was added prior to casting the gel.

### 2.2. Sector Phantom

To investigate the impact of tissue susceptibility on the movement of substances with varying diffusivity, a cylindrical phantom comprising eight sectors was constructed. Six of the sectors were filled with agar gels that had undergone varying degrees of sucrose fracturing. Furthermore, [18F]FDG was incorporated into the gels in two sectors. 

### 2.3. Tumor Phantom

In addition to spherical phantoms, we constructed a phantom consisting of two concentric spheres to simulate an FDG-avid tumor embedded in healthy tissue. The solid inner sphere represents the tumor and was filled with agar gel mixed with [18F]FDG. The surrounding cavity was filled with agar gel with a different sucrose fraction in order to simulate a different diffusivity compared to the tumor.

### 2.4. Phantom MR and PET Measurements

All measurements were made on a 3T hybrid PET/MRI scanner (Biograph mMR, Siemens Healthineers, Erlangen, Germany). T2-weighted images were obtained using a TSE sequence with $T_{R}=6410\mathrm{ms}$, $T_{E}=93\mathrm{ms}$, and a flip angle of 150°. Diffusion-weighted images were obtained using the Readout SEgmentation Of Long Variable Echo-trains (RESOLVE) sequence with $T_{R}=5230\mathrm{ms}$, $T_{E}=56\mathrm{ms}$, 2 averages, and a flip angle of 180°. For the DWI measurements, we used a slice thickness of 5 mm with a slice distance of 6 mm and b-values of 50 s/mm^2^ and 800 s/mm^2^. The PET acquisition time was extended to the MR measurements. We used the built-in 3D-ordered subset expectation maximization (OSEM) PET reconstruction with MR-based attenuation correction. 

### 2.5. Patients

A total of twelve consecutive patients who had undergone initial staging and who had been histologically confirmed to have bronchial carcinoma underwent PET/MRI. The mean age of the patients was 64.9 years (range: 42–81), with four females. In all cases, the diagnosis was confirmed by histology. The retrospective analysis was approved by the local ethics committee (480/20).

### 2.6. PET/MR Examinations

Patients were scanned at the same PET/MR scanner after the injection of [18F]FDG 60 min prior to the examination. Fasting was advised for at least four hours prior to injection. The injected activity was adapted by body weight (310 to 340 MBq). In total, 10 mg furosemide was used for faster renal clearing of [18F]FDG. Gadobutrol was used as a contrast agent (Gadovist, Bayer AG, Leverkusen, Germany) and adapted by body weight (1 mL/10 kg body weight).

For attenuation correction, we used a volumetric interpolated breath-hold examination (VIBE) protocol with a Dixon reconstruction and inline creation of a five-compartment (air, lung tissue, fat, soft tissue, and bone) µ-map based on an anatomical atlas [27,28]. Diffusion-weighted images were measured with a single-shot 2D monopolar echo-planar imaging sequence (EPI) and b-values of 50 s/mm^2^ and 800 s/mm^2^ were used. 

### 2.7. Data Preparation and Co-Registration

For the co-registration of the images, we used an automatic 3D elastic co-registration with a volume-preserving constraint built in the MR Multiparametric Analysis research application (Version 1.2.1, Siemens Healthineers, Erlangen, Germany) [29]. The T2-weighted images were used as the reference image for phantom measurements and ADC maps were registered.

For patients, the post-contrast water image of the DIXON sequence (wDIXON) and DWI images were loaded into the software, obtaining volume-preserving 3D elastic registration as previously described [30,31] with the original image output from the scanner. The wDIXON image was used as a static image and the low-b-value diffusion-weighted image to be registered. The moving image was automatically resampled by the toolbox to match the dimensions of the static image. The deformation field obtained by the elastic registration was then applied to the apparent diffusion coefficient (ADC) map. Registration results were exported for comparison evaluation. The process of image co-registration is illustrated in Figure S1.

### 2.8. Image Evaluation

Three readers with a minimum of six, eight, and more than twenty years of experience in reading multiparametric imaging evaluated the images and were blinded to the original and transformed image datasets. The test series consisted of 12 examinations. 

Each dataset consisted of six image series, wDIXON, diffusion-weighted b50, and ADC map, for both registered and non-registered image datasets. Furthermore, the reader was furnished with a “subtracted” series, which contains the magnitude of the difference calculated by the subtraction of the registered ADC map from the original ADC map. This series serves as a visual representation of the areas most affected by the registration algorithm, providing a useful guide for the eye. The series are presented in a side-by-side format, with a commercial reading software (syngo.via MM Oncology Workflow, Siemens Healthineers, Erlangen, Germany) used to display them. An additional colored overlay is also used, showing the diffusion-weighted images and ADC maps on the wDIXON for both series. Linked scrolling and correlating cursors were employed to facilitate the evaluation of anatomical accuracy. The accuracy of the registration was evaluated for a subset of organs and anatomical structures and scored on a Likert scale from 1 (poor fitting) to 5 (ideal fitting). The evaluation was based on the identification of anatomical landmarks, which were defined for each region. The abdomen was evaluated using eight landmarks: the contour of the liver, spleen, pancreas, left and right kidneys, and gall bladder and contour of the skin and vertebral bodies. The thorax was evaluated using six landmarks: the aortic arch, tumor, contour of the left ventricle, contour of the skin, inner contour of the thoracic wall, and vertebral bodies. The process of image evaluation is illustrated in Figure S2.

### 2.9. DWI and PET Registration

In a second analysis, the combination of rigid and non-rigid 3D registration with a volume-preserving constraint was repeated to correct PET images. Scatter plots were obtained in the Multiparametric Analysis software (version 1.2.1) for corresponding ADC and PET data for the volume of interest derived from the manual segmentation of the thoracic tumors. 

### 2.10. Evaluation of Registered Datasets

The assessment of the improvement in spatial accuracy of parametric maps before and after image co-registration by visual comparison is inherently subjective. For several pathologies, a correlation of ADC and standardized uptake values based on body weight (SUVbw) is proposed in [32,33,34]. However, it is important to note that these correlations may not be universally applicable to all tumor and tissue types. In particular, discrepancies between the two parameters are of significant interest when conducting a voxel-wise analysis. Consequently, a simple correlation analysis between SUV and ADC does not adequately reflect the quality of the image fusion. Therefore, we postulated that a substantial spatial mismatch could potentially lead to clinically implausible combinations of ADC and SUVbw values, suggesting the absence of a biologically plausible correlate. The volume of those regions exhibiting biologically implausible values could therefore be employed as an indicator of the accuracy of the co-registration. The influence of co-registration on the statistical distributions of the parametric ADC and SUVbw values in tumor volumes of interest (VOIs) was evaluated. Scatterplots were generated using the ADC and spatially corresponding SUVbw values within the VOIs of the registered and original datasets. The VOIs were delineated on the T1w wDIXON images, which served as the static image for co-registration of the DWI and PET images. In accordance with published data [16,34,35,36,37,38,39], we deemed the following combinations on the scatterplots to be clinically less plausible:

- ADC < 300 mm^2^/s in combination with an SUVbw > 4.
- ADC > 1600 mm^2^/s in combination with an SUVbw > 4.
- ADC > 300 mm^2^/s and < 1600 mm^2^/s in combination with an SUVbw < 1.

ADC values below 300 mm^2^/s are presumably found in low-density “air-like” tissue like lung parenchyma or fat, and thus we expect no or only minimal [18F]FDG uptake. Thus, a correlating SUVbw > 4 suggests a mismatch as it is a less plausible combination of ADC and SUV. On the other hand, very high ADC values suggest fluids or necrosis, and thus again only minimal [18F]FDG uptake is expected and SUV more than 4. Finally, intermediate ADC between 300 and 1600 suggests that there is some kind of tissue/tumor, and thus we expect at least some [18F]FDG uptake and very low SUVs below 1 also seem less plausible. 

To quantify the influence of the co-registration, the total number of voxels with less plausible combinations of ADC and SUVbw before and after co-registration were counted. We considered a decrease in the number of less plausible voxels after co-registration as an improvement in spatial accuracy. In addition, we evaluated the statistical moments of the distributions to further support our theory. 

### 2.11. Statistics

All values are reported as the mean ± standard deviation (SD) if not stated otherwise. A one-way analysis of variance (ANOVA) with repeated measurements was used to compare phantom measurement results before and after registration in comparison to the expected ADC. A Wilcoxon signed rank test was used for comparisons of Likert scale ranks. All statistical tests were performed using the SciPys statistics module (SciPy Version 1.7.1, Python 3.8.5) [40]. Scatter plot images were generated with DataGraph (Version 5.2, Visual Data Tools).

## 3. Results

### 3.1. Workflow

The workflow entails the labeling of the utilized sequences and attenuation-corrected PET images, respectively. In a second step, a combination of rigid and non-rigid three-dimensional registration with a volume-preserving constraint is applied using an anatomical sequence as a base, prior to the delineation of regions of interest (ROIs) in the tumor or phantom region. Finally, the backpropagation of the voxel-wise combinations of ADC and PET values to the anatomical sequence allows for the visualization of color-coded thresholds of ADC and SUV combinations. This workflow has been successfully applied to all phantom measurements and patient examinations.

### 3.2. Sector Phantom

**Figure 1:**
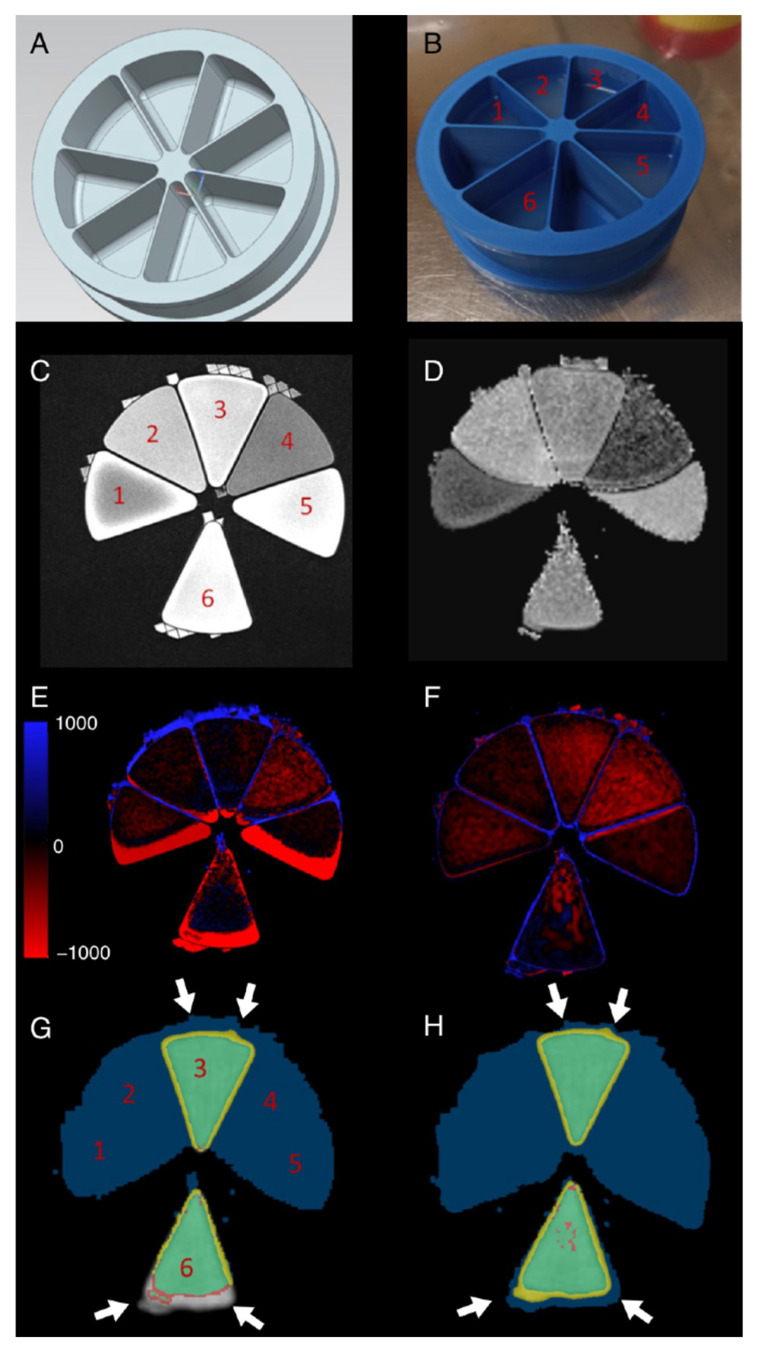
The sector phantom study with the 3D model for printing (**A**); an image of the phantom with agar gel that filled sectors 1–6 with varying sucrose fractures (**B**). In addition, [18F]FDG was added to the gels in sectors 3 and 6. A T_2_-weighted image (**C**), ADC map derived from RESOLVE acquisition (**D**), and T_2_-weighted background image with colored ADC map overlay before registration (**E**) and after registration (**F**). Blue and red coloring indicates deviations from the expected and the measured ADC values. Overlays (**G**,**H**) show the fused ADC (blue) and PET images (green) before (**G**) and after registration (**H**) with improved overlay especially at the borders (white arrows).

Subsequently, measurements were conducted following the successful generation of the sector phantom (Figure 1A,B). A comparison of the T2-weighted image (Figure 1C) with the ADC map (Figure 1D,E) reveals that the latter exhibits geometric distortions. The greatest distortions are observed at the interface between tissue and air, due to the differing magnetic susceptibilities. A comparison of the warp effect on the transitions from sectors 1–2, 2–3, 3–4, and 4–5 revealed that tissues with different diffusivity did not exhibit significant distortions (Figure 1D). The described 3D fully elastic co-registration algorithm was employed to restore the geometric shapes and area, resulting in a high degree of agreement with the T2w image (Figure 1F) and an improved overlay of ADC and PET images (Figure 1G–H). A comparison of the area with a signal before and after registration for all six sectors revealed an improvement in accuracy resulting from the registration (Table 1). No significant differences were observed in the measured ADC values between unregistered and registered datasets in comparison to the expected values (F = 1.12, *p* = 0.34).

### 3.3. Sphere and Tumor Phantoms

**Figure 2:**
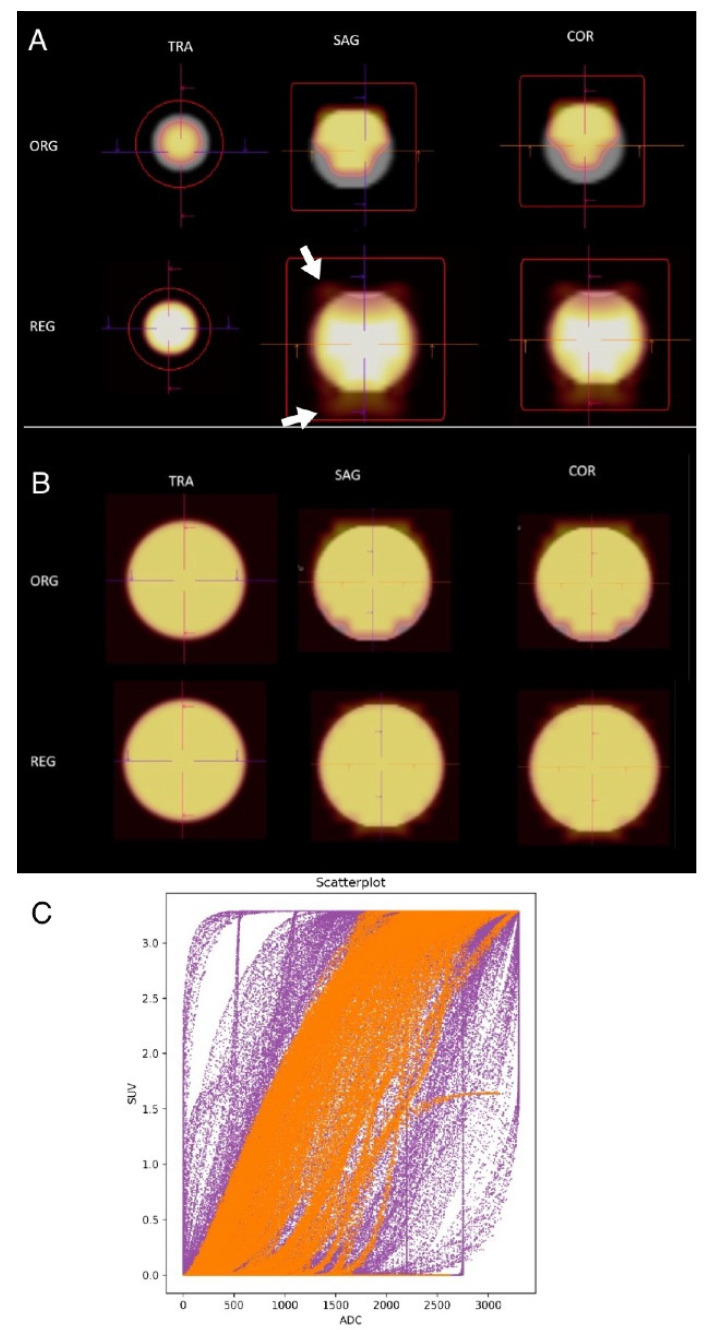
Sphere phantoms of different sizes were used to test the algorithm regarding dependencies on lesion and slice thickness. (**A**) An agar gel-filled sphere 20 mm in diameter was used for T2-weighted measurements (1 mm slice thickness) and ADC images (colored overlay, 5 mm slice thickness, 20% distance factor) were registered to the T2 images. The top row represents the original (ORG), unregistered images in transversal (TRA), sagittal (SAG), and coronal (COR) orientation; the lower row shows the registered images (REG). While the original images show misregistration in the periphery and the lower areas of the sphere, the registered images show misregistration outside of the contours of the T2 image (white arrows). (**B**) An agar gel-filled sphere 20 mm in diameter was used for T2-weighted measurements (1 mm slice thickness) and ADC images (colored overlay, 5 mm slice thickness, 20% distance factor). The registered images show improved overall alignment both to the unregistered original overlay as well as compared to the smaller sphere in (**A**). (**C**) A scatter plot of single-voxel representations of ADC values (*x*-axis) and SUV (*y*-axis). The purple pixels represent the values before the registration of the sphere shown in (**B**) (ORG), and orange pixels represent after registration (REG). Especially, a shift of voxels away from very low ADC/high SUV values and very high ADC/SUV values can be observed.

To investigate the necessary lesion size applicable for the registration regarding slice thickness, we created several sphere phantom measurements with different sizes. In analogy to our clinical protocol, we sampled 5 mm slices with a 6 mm slice distance for multiple sets of spheres with increasing diameter. We identified a threshold with the lesion diameter of at least four times the slice thickness to avoid registration artifacts with 3D elastic co-registration. A sphere 20 mm in diameter produced blurry artifacts at the borders of the lesion after registration when imaged with a typical clinical examination protocol for DWI with a 5 mm slice thickness and 20% distance factor (Figure 2A). This was not the case in larger spheres (Figure 2B) and we identified a lesion size of four times the slice thickness as a threshold for a feasible 3D elastic co-registration. The analysis of single voxels as outlined in the scatter plot in Figure 2C revealed a shift of voxels away from very low ADC/high SUV values and very high ADC/SUV values.

**Figure 3:**
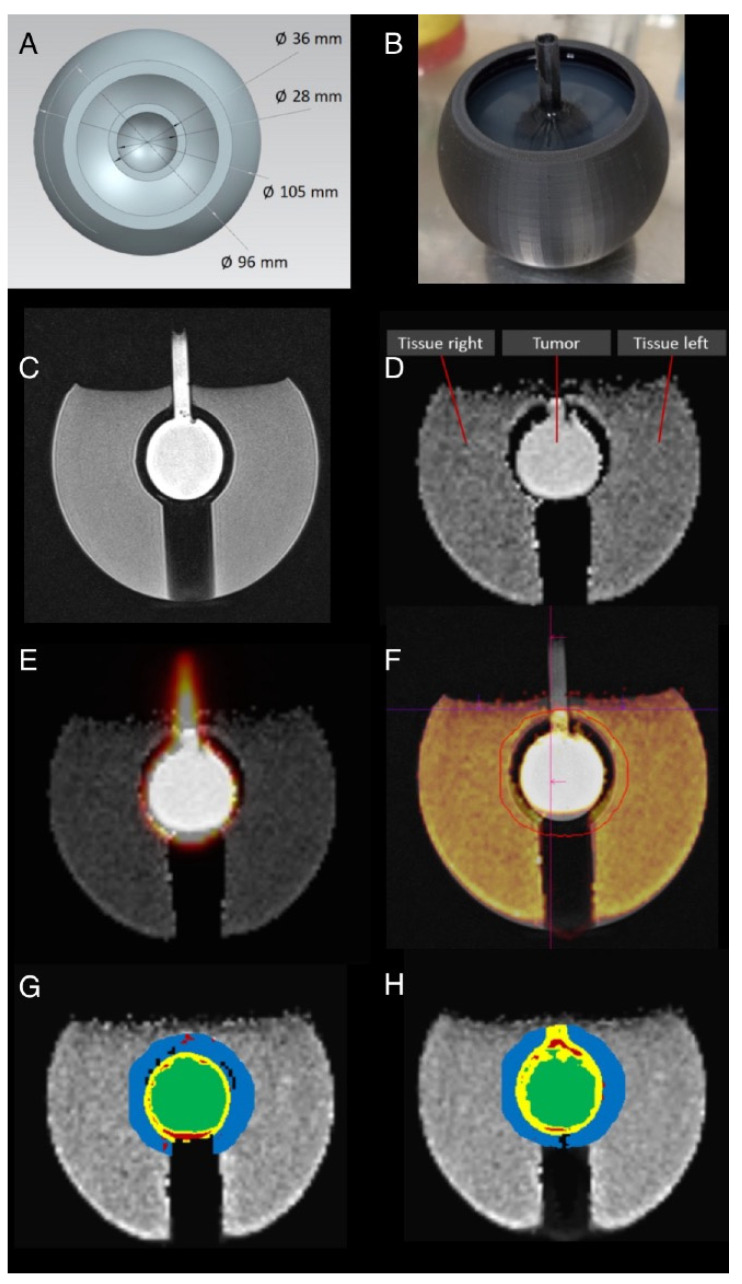
Tumor phantom study with 3D model for printing (**A**); image of phantom with agar gel-filled outer layer (**B**). In addition, [18F]FDG was added to central compartment (“tumor”). T2-weighted image (**C**), ADC map derived from RESOLVE acquisition (**D**), and PET image in yellow colors overlayed on ADC map before registration (**E**) and after registration (**F**) with T2-weighted background image with colored ADC map overlay. Drawn volume of interest is indicated as red line. Back mapping of the scatterplot before (**G**) and after registration (**H**) from ADC and the PET-image are given.

The tumor phantom measurements with a central FDG-containing lesion adjacent to a tissue equivalent showed some susceptibility artifacts at the surface of the phantom (Figure 3), to resemble body surfaces. Especially in the bottom area of the inner sphere, the ADC registration seems to improve the spatial agreement to the T2-weighted image (Figure 3F). This is also acknowledgeable in the back mapping of the scatterplot from ADC and PET-images (Figure 3G,H).

### 3.4. Thoracic Tumor Segmentation and Registration

**Figure 4:**
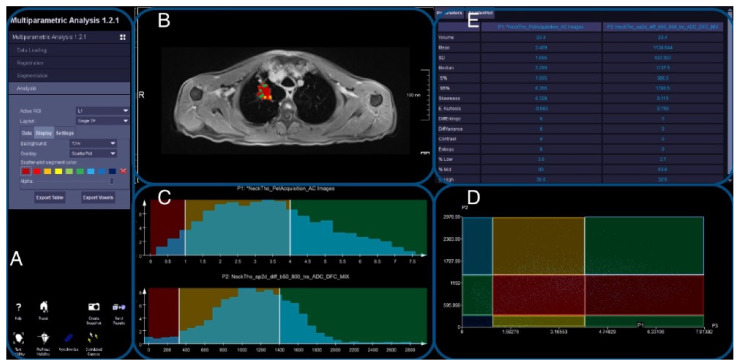
The overview of the prototype workflow: After co-registration, the overlay and back mapping of the scatterplot from PET and ADC can be selected (**A**) and mapped to the T1w image in (**B**). (**C**) illustrates the scatter plots for SUV (**top**) and ADC values (**bottom**, non-matched color coding). Here, thresholds can be defined, and regions for combinations of ADC and SUV values can be color-coded and visualized in a combined scatter plot (**D**), which are represented in the back mapping (**B**). Descriptive analyses are visible in (**E**). In (**D**), green areas represent implausible combinations of ADC and [18F]FDG, dark blue represents air with very low ADC and SUV values, light blue characterizes fluid areas with very low SUV and high ADC values, and yellow and orange tones represent tissue with moderate FDG uptake and either very low or high ADC values. Red tones signify solid tissues with low ADC values and moderate-to-high FDG uptake, representing the tumor (core).

**Figure 5:**
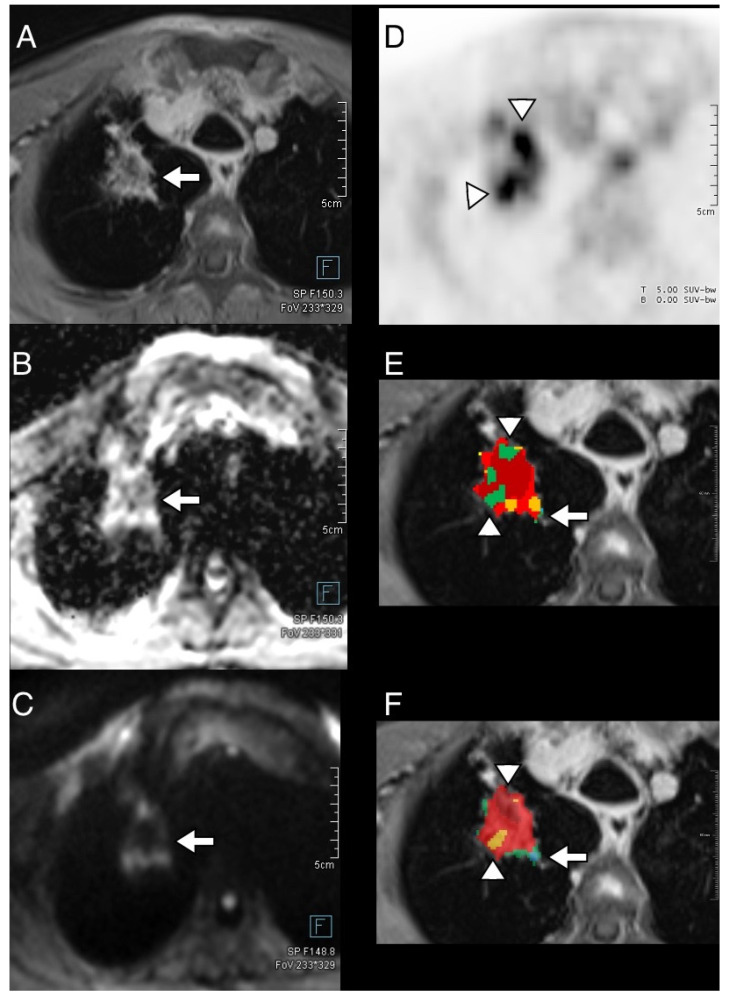
An example for the tumor segmentation of a bronchial carcinoma (NSCLC) in the right lung in a 64-year-old patient with T1w DIXON (**A**), ADC map (**B**), and b800 from diffusion-weighted imaging (**C**) with the indication of the tumor (arrows). Regional differences in FDG uptake can be noted in (**D**) with higher focal uptake (arrowheads). PET and ADC are co-registered, and a volume of interest is defined on the T1w image. Color-coded back mapping of the scatterplots (see Figure 6) is shown for unregistered (**E**) and registered (**F**) datasets. Note the substantial improvement in the co-registration with regard to the areas with high FDG uptake (arrowheads, yellow and dark red areas in (**F**), same color coding as in Figure 4) and the improved delineation of tumor/air boarders in green (arrows in (**E**,**F**)) that represent implausible combinations of ADC and SUV values.

Tumor segmentation was feasible for all clinical examinations. The overall visual impression for all thoracic examinations showed a significant improvement for DWI and morphological landmark alignment in the 3D-registered datasets as seen by all readers (original vs. 3D-registered dataset: 4.3 ± 0.2 vs. 4.6 ± 0.2 points, *p* = 0.009). The most pronounced differences were noted for the chest wall (*p* = 0.006), followed by the tumor (*p* = 0.007) and the skin contour (*p* = 0.014). This is also reflected by an overall average increase in alignment for the chest wall and the tumor for the average reader (+2.0 points for the chest wall, equal to 18.0% mean improvement between readers; +1.42 points for the tumor, equal to 18.4% mean improvement, respectively). Significant improvements were also noted for the contour of the aortic arch (*p* = 0.01), while the contour of the left ventricle and the vertebral body contours were not altered significantly (*p* = 0.06 and 0.6, respectively). An example for the tumor segmentation is given in Figure 4 and a patient example is given in Figure 5.

### 3.5. Plausibility of DWI and PET Images

**Figure 6:**
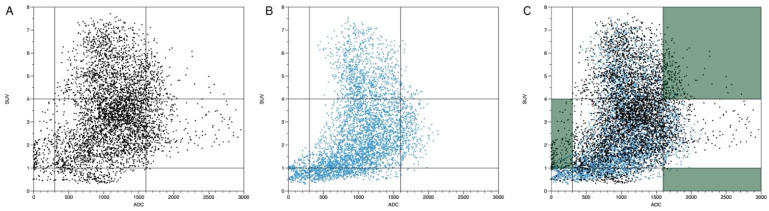
A scatterplot demonstrating the ADC and SUV combinations (same data as in Figure 5) with the voxel distribution before (**A**) and after (**B**) co-registration. The combined demonstration of (**A**,**B**) in (**C**) demonstrates the voxel changes after registration. Note the reduced width of the scatter plot and the reduction in voxels in the area of clinically implausible values with low ADC values < 300 and SUV between 1 and 4, as well as ADC > 1600 and SUV > 4.

In a second step, the registration matrix for registered DWI and wDIXON images was used to additionally register PET images from the dedicated examinations. Scatter plots of lesion VOIs for ADC and SUVbw values were segmented before and after co-registration. For all datasets combined, a total of 53233 voxels were registered. Of those, 7390 voxels before registration and 5537 voxels after registration were considered less plausible. On a per-lesion basis, the reduction in less plausible values ranged from 1 to 9% regarding the total number of voxels. In total, the number of plausible voxel combinations of ADC and SUVbw could be increased by 25% in thoracic images after co-registration. An example for the scatter plots of ADC/SUV data is given in Figure 6. The distribution of the individual voxels in the ADC/SUV scatterplot shifted after the optimized registration. The mean value for ADC and SUV was affected very little (−0.6% ± 13.2% for ADC and −0.7% ± 7.2% for SUV).

## 4. Discussion

While simultaneous acquisitions in PET/MR address many previous issues with the overlay of PET and MRI images, there are still unresolved issues regarding the co-registration of PET and MRI images when considering different MRI sequences. In this study, we addressed these issues using a rigid and elastic co-registration algorithm to investigate the voxel-wise improvement in PET and ADC in one of the most challenging regions for PET/MR, the thorax. The results demonstrated that image registration in both phantom and patient data led to both visual and quantitative improvements. 

The proposed workflow can be applied in all cases, and the generation of the registered datasets can be completed in a reasonable time frame that can be integrated into clinical practice. 

The phantom experiments served to reiterate the clinically relevant issue of geometric distortions in ADC maps (Figure 1D). Local field inhomogeneities resulting from disparate magnetic susceptibility of the tissue exert the most significant influence on the spatial accuracy of diffusion-weighted images and the derived ADC map. In particular, at transitions between air and tissue, the distortions can be highly pronounced, substantially impairing image analyses. The artifact warps the boundaries along the phase encoding direction, thereby leading to a misrepresentation of the geometric shape and area. These phenomena impede the precise voxel-wise registration of anatomy, PET, and ADC. The employed co-registration methodology demonstrated enhanced overlay of ADC and PET measurements on the anatomical T2w image in phantom measurements. It is noteworthy that the enhanced overlay was contingent upon the lesion size in relation to the utilized slice thickness. This interplay between ADC and SUV values has been previously highlighted in in vivo measurements before [16], and is of critical importance when applying the aforementioned registration algorithm. 

While numerous studies have examined the relationship between SUV and ADC in a multitude of cancers, only a select few have investigated this relationship at the voxel-wise and histogram level. Wongsa et al. analyzed the maximum coefficient of variation for SUV and ADC values derived from simultaneous PET/MRI examinations of head and neck cancer patients. The researchers observed a maximum coefficient of variation of 13.9% and 9.8% for both parameters in phantom and in vivo studies, respectively. They concluded that low variability was achieved after the implementation of median noise filtering and the N4 bias field correction algorithm [23]. However, no registration method was employed for ADC or PET images. Orsatti et al. employed volumetric histogram analyses in pediatric sarcoma patients to investigate the relationship between ADC and SUV values [41]. The authors proceeded to collect combinations of SUV and ADC values. Subsequently, they conducted volumetric histogram analyses, which led them to conclude that ADC and SUV values are dependent biomarkers in pediatric FDG-avid sarcomas. A registration step was not included prior to lesion segmentation. Additionally, Meyer et al. [42] investigated the relationship between ADC and SUV values in cervical cancer using a histogram analysis, yet did not employ a dedicated registration step from PET/MRI-derived datasets in eighteen patients. In a recent study, Chaika et al. and Männlin et al. employed a voxel-wise approach to investigate the relationship between SUV and ADC values in a small number of neuroblastoma and rhabdomyosarcoma patients before and after chemotherapy [24,25]. They demonstrated that the clustering of SUV and ADC values differs between patients who responded to treatment and those who did not. The feasibility and clinical potential of a voxel-wise approach are demonstrated by this approach. Other attempts have been made to improve the registration of PET and MRI datasets. However, these mainly focus on PET data derived from PET/CT and a separately acquired MRI, which by itself introduces significant motion and overlay challenges [43,44]. 

The analysis of the plausibility of SUV and ADC combinations remains a crucial aspect of this initial analysis. Tumor heterogeneity is a well-known factor relevant for prognoses and aggressiveness [45]. Therefore, the precision of the overlay of the different datasets is of paramount importance to derive meaningful combined data and to avoid erroneous conclusions based on the combination of voxels. In our study, the number of voxels with clinically implausible combinations of SUV and ADC (e.g., those containing air in one of the two examinations) was reduced after applying the registration algorithm. This approach may prove useful in future studies aimed at preserving a biologically meaningful interpretation of tumor heterogeneity. While the definition of thresholds for plausible and less plausible combinations of SUV and ADC values must be established for each disease in question [24] and the suggested values are open to debate, it appears that the registration process has the potential to reduce values of less plausible combinations in all examinations while maintaining the average values. The minimal, unnoticeable alterations can be attributed to the reduction in values associated with less plausible combinations. This is of importance regarding the clinical implementation of this approach.

This study is limited by the small number of patients and the absence of a clinical evaluation of the presented data. However, this represents a preliminary attempt to improve the voxel-wise overlay of SUV and ADC from phantom to patient measurements, with the aim of enabling the analysis of data at the single-voxel level. Further studies are now required to assess the clinical importance of this approach. The objective was to employ a methodology that was simple and straightforward to implement, and whose steps were readily comprehensible. Consequently, a deep learning algorithm was not utilized at this juncture. Additionally, we do not provide a histopathological correlation, which would be beneficial for further investigations. However, in clinical practice, obtaining such a correlation is challenging. Consequently, we initially conducted phantom studies, which serve as a reference standard for the expected ground truth, although they are not equivalent to in vivo studies on tumor heterogeneity. An additional challenge that is beyond the scope of this initial analysis, but an important issue that must be considered for future analyses, is the improvement in inherent motion correction in the lungs and heart. This is of particular relevance for examinations of the thorax and upper abdomen. This represents a continuing area of investigation [46].

## 5. Conclusions

This study presents a novel approach to further investigate the relationship of regional tumor heterogeneity. The application of a combined rigid and non-rigid 3D registration algorithm is utilized to improve the voxel-wise correlation of PET and MRI-derived values in order to enhance simultaneous PET/MRI acquisitions. The results from phantom and patient data demonstrate the robustness of the utilized registration algorithm for multiparametric and multi-model imaging, which can thus be used as a basis for future prospective clinical studies. 

## Figures and Tables

**Table 1:** Quantified volumes of interest from the six sectors of the phantom. Both area and ADC values are given for expected, unregistered, and registered measurements. No significant differences were observed in the measured ADC values between unregistered and registered datasets in comparison to the expected values (F = 1.12, *p* = 0.34).

Sector	Area [cm^2^]	Area [cm^2^] ± SD Unregistered	Area [cm^2^] ± SD Registered	Expected ADC [mm^2^/s] ± SD	Unregistered ADC [mm^2^/s] ± SD	Registered ADC [mm^2^/s] ± SD
1	16.2	15.6 ± 1.6	15.1 ± 1.6	800 ± 130	730 ± 80	660 ± 70
2	16.2	21.2 ± 2.2	14.7 ± 1.5	1400 ± 100	1310 ± 140	1330 ± 70
3	16.2	22.6 ± 2.3	13.0 ± 1.4	1110 ± 110	1120 ± 120	1130 ± 70
4	16.2	21.1 ± 2.2	14.9 ± 1.5	800 ± 130	550 ± 200	530 ± 100
5	16.2	15.1 ± 1.6	16.5 ± 1.7	1400 ± 100	1370 ± 80	1350 ± 40
6	16.2	9.8 ± 1.0	17.1 ±1.8	1110 ± 110	1260 ± 180	1230 ± 80

## Data Availability

The data that support the findings of this study are available from the corresponding author upon reasonable request.

## Supplementary Materials

The following supporting information can be downloaded at: https://www.mdpi.com/article/10.3390/diagnostics14161787/s1, Figure S1: Flow chart for image registration in patients. Figure S2: Hanging layout for reading sessions.
