# Supplementary material for: From Phantoms to Patients: Improved Fusion and Voxel-Wise Analysis of Diffusion-Weighted Imaging and FDG-Positron Emission Tomography in Positron Emission Tomography/Magnetic Resonance Imaging for Combined Metabolic–Diffusivity Index (cDMI)
Source: Diagnostics (Basel). 2024 Aug 16;14(16):1787. doi: 10.3390/diagnostics14161787 (PMC11353375; doi:10.3390/diagnostics14161787)
Supplement: Supplementary file 1 [file diagnostics-14-01787-s001.zip › diagnostics-3118652-supplementary.pdf]

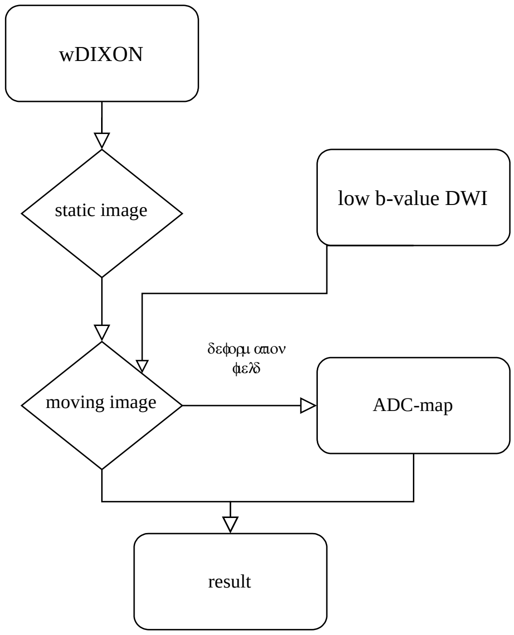

Figure S1: Flow chart for image registration in patients. The post contrast water image of the DIXON sequence (wDIXON) is used to generate a static image and the low b-value diffusion-weighted image to be registered. The moving image is automatically resampled by the toolbox to match the dimensions of the static image. The deformation field obtained by the elastic registration is then applied to the apparent diffusion coefficient (ADC) map. A volume-preserving 3D elastic registration algorithm was applied. Registration results were exported for comparison evaluation.

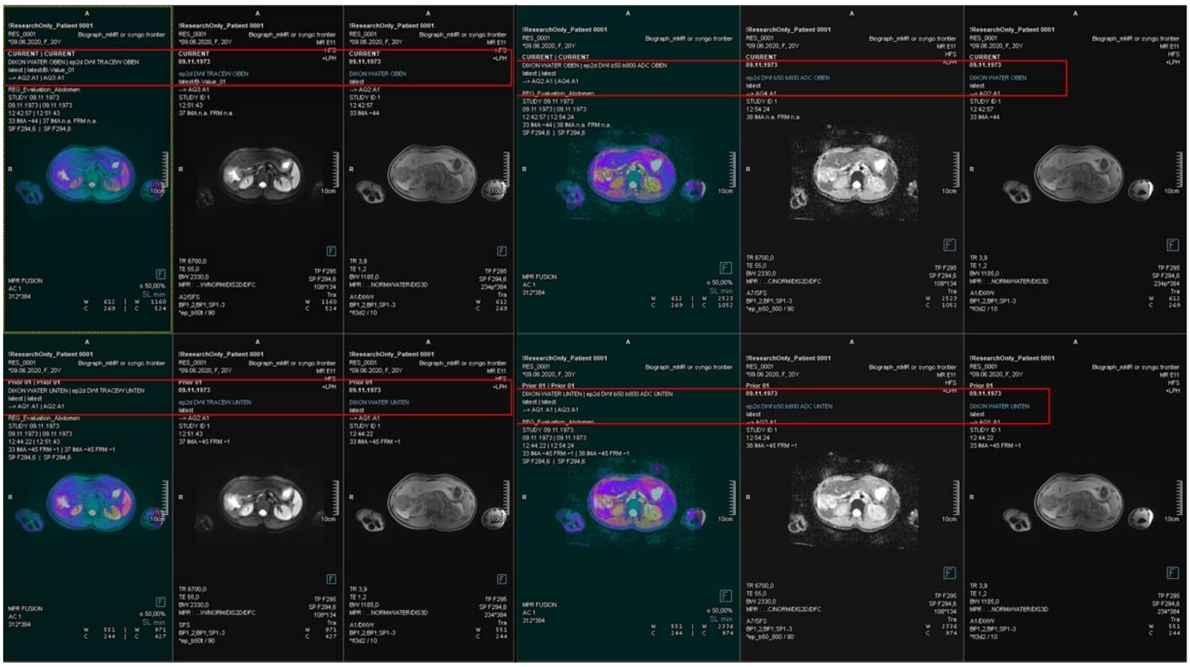

Figure S2: Hanging layout for reading sessions. Top row: original datasets (no registration algorithm applied, from left to right: fusion of low b-value diffusion-weighted image and DIXON water images, individual images; fusion of ADC and DIXON water images, individual images) and the co-registered datasets in the bottom row in the same order.
